# Surgical treatment for rectal cancer with abnormally expanded inferior mesenteric vein resulting from pancreatic arteriovenous malformations

**DOI:** 10.1186/s40792-015-0021-9

**Published:** 2015-02-25

**Authors:** Hiroshi Tanabe, Tsunenobu Takase, Takahiro Inaishi, Mariko Masubuchi, Naohiro Nomura, Arihiro Shibata, Toyohisa Yaguchi, Eiji Ohnishi, Norio Okumura, Shinya Koike, Kouichirou Tagami

**Affiliations:** Department of Surgery, Atsumi Hospital, 1-1 Akaishi, Kanbe-cho, Tahara, Aichi 441-3415 Japan; Department of Surgery, Kainan Hospital, 396 Minamihonden, Maegasu-cho, Yatomi, Aichi 498-8502 Japan

**Keywords:** Pancreatic arteriovenous malformation, Rectal cancer, Inferior mesenteric vein, Arteriovenous malformation, Distal pancreatectomy, Laparoscopic surgery, Laparoscopic abdominoperineal resection

## Abstract

A 52-year-old Japanese man presented for evaluation and treatment of rectal cancer. Screening computed tomography revealed pancreatic arteriovenous malformations (P-AVMs) and abnormally expanded inferior mesenteric vein (IMV) that resulted from P-AVMs. One-stage surgery for rectal cancer was dangerous so we first performed distal pancreatectomy to cure P-AVM and thus normalize the abnormally expanded IMV. After the operation, the IMV was occluded by the thrombi, and then the IMV became normal. We could perform safely radical laparoscopic surgery for rectal cancer. This is the first case report of P-AVMs combined with rectal cancer.

## Background

Pancreatic arteriovenous malformation (P-AVM) is a vascular anomaly in which blood flows from the arterial system directly into the portal venous system without passing through the capillaries in the pancreas [1]. It is a rare disease and less than 100 cases have been reported in the English language literature.

The current report describes a patient with abnormally expanded inferior mesenteric vein (IMV) resulting from P-AVMs, which was revealed during preoperative screening computed tomography (CT) for rectal cancer. We first performed distal pancreatectomy to cure P-AVMs, and then the abnormally expanded IMV became normal. Subsequently, we could perform laparoscopic abdominoperineal resection safely.

## Case presentation

**Figure 1:**
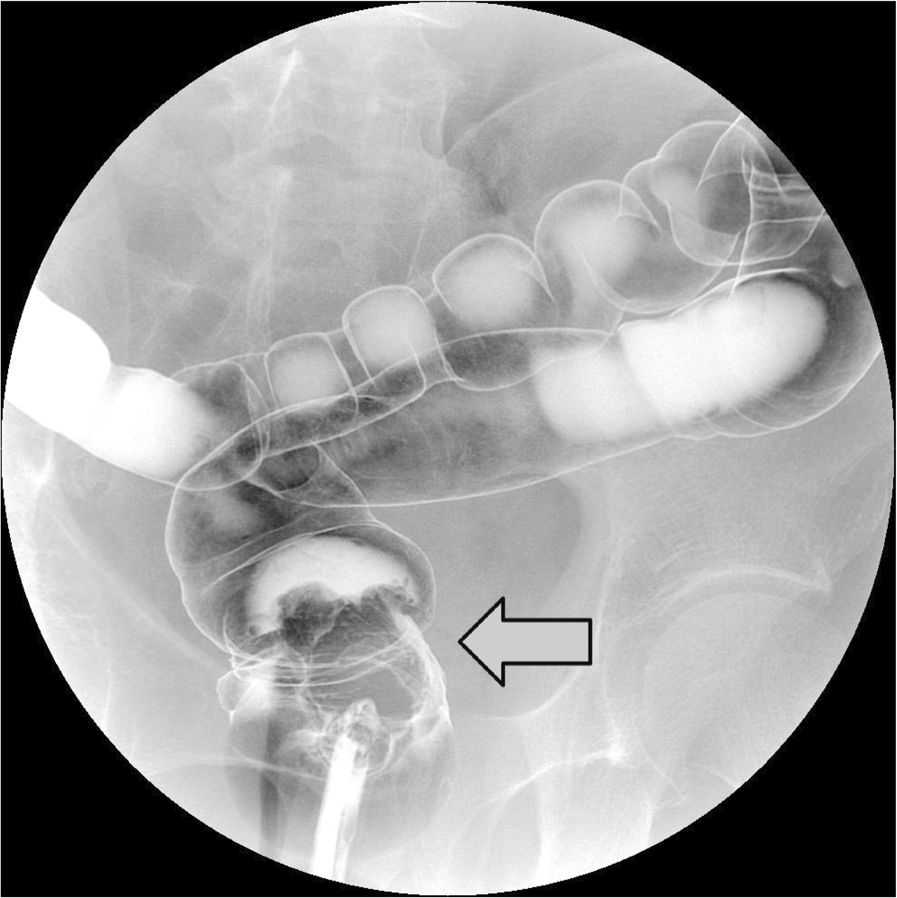
**Barium enema showed a filling defect at the rectal ampulla (arrow).**

**Figure 2:**
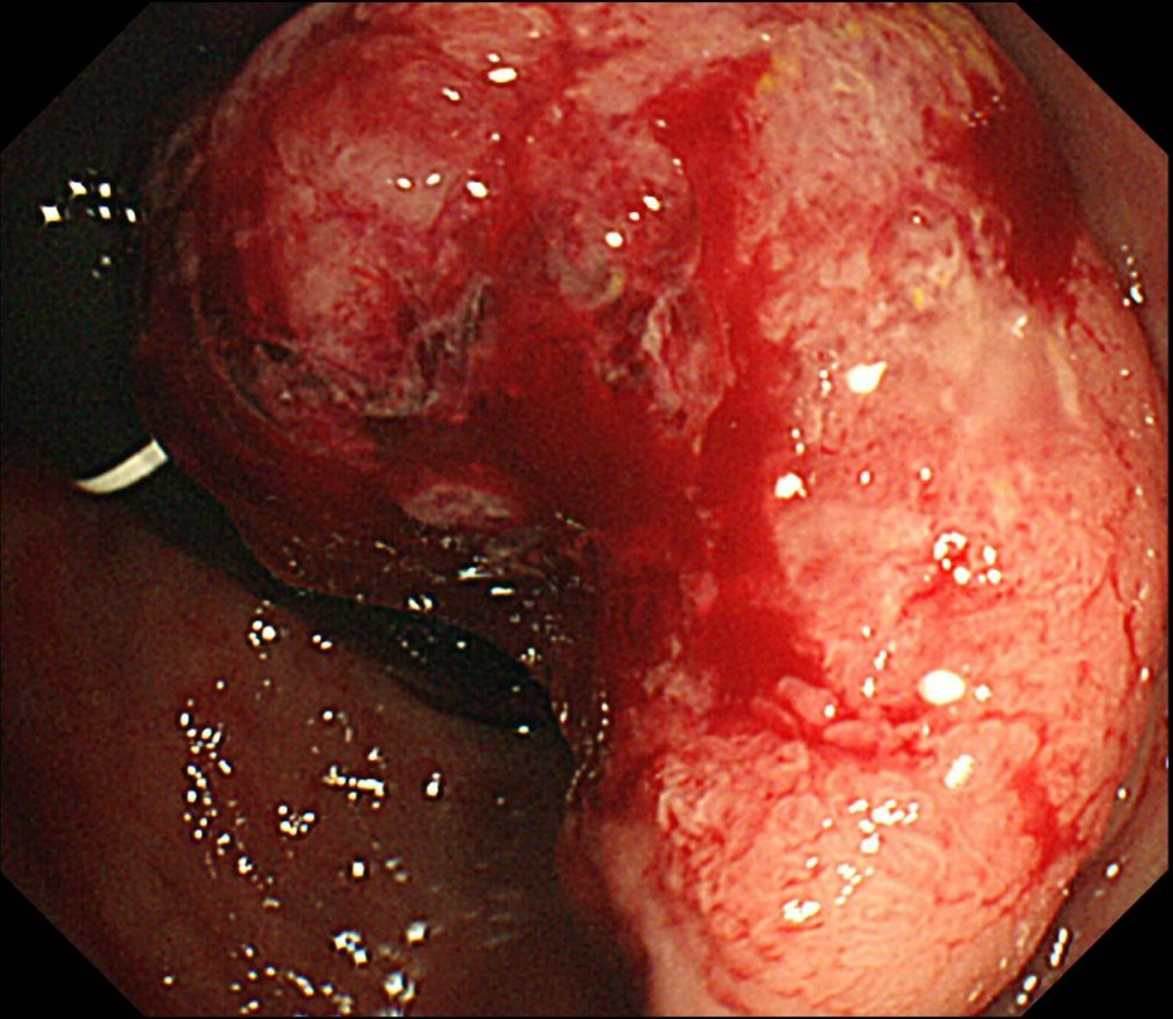
**Colonoscopy revealed a type 2 tumor localized in the lower rectum.**

**Figure 3:**
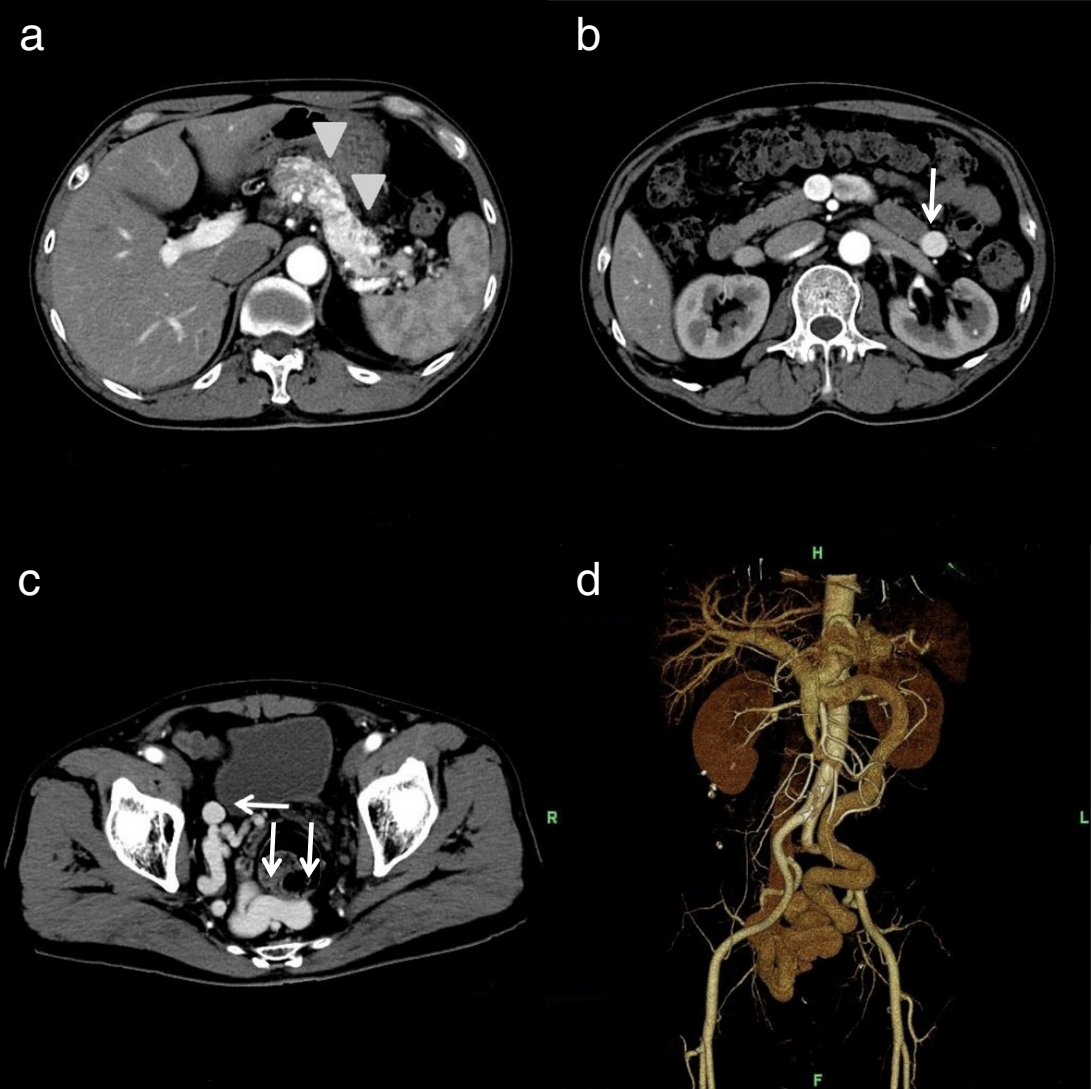
**Hypervascular spots and abnormally expanded IMV. (a)** Preoperative CECT revealed hypervascular spots in the pancreatic body and tail (arrowhead). **(b)** Abnormally expanded IMV (arrow). **(c)** Abnormally expanded IMV (arrow) in the pelvic space. **(d)** 3D reconstruction.

**Figure 4:**
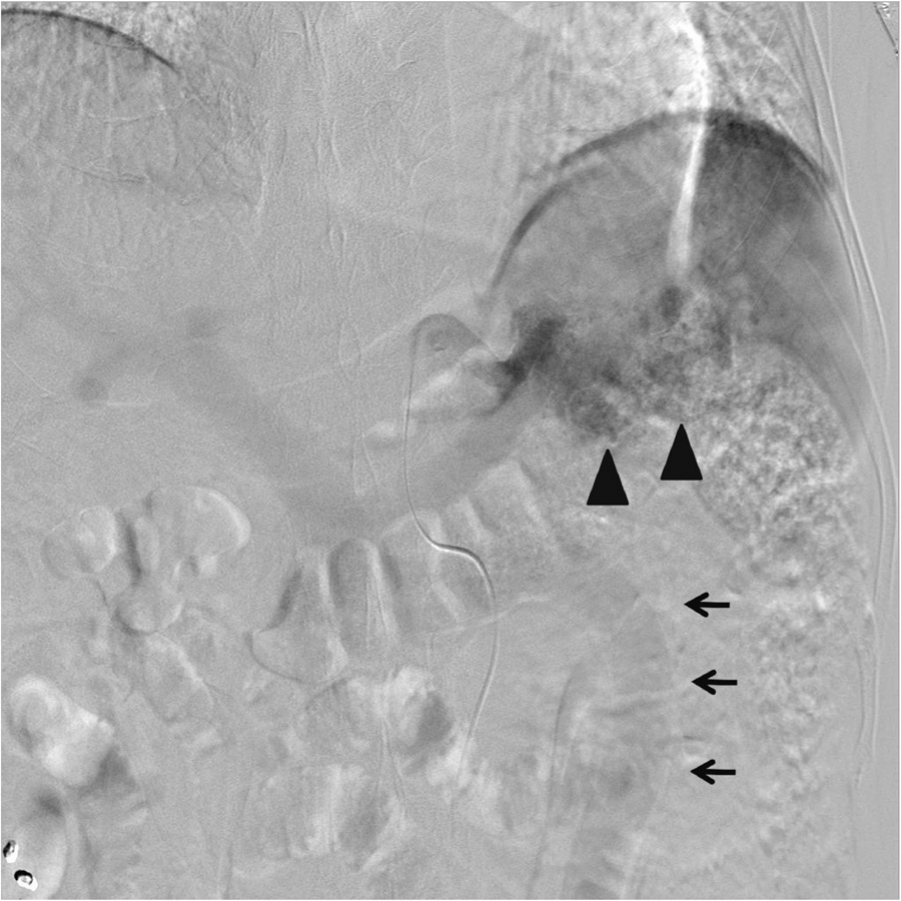
**Racemose vascular networks in the pancreas, early venous return, and abnormally expanded IMV.** Preoperative angiography confirmed racemose vascular networks in the body and tail of the pancreas (arrowhead), and early venous return to the portal venous system and the abnormally expanded IMV (arrow).

A 52-year-old Japanese man presented at our hospital for evaluation and treatment of rectal cancer. He had been diagnosed with rectal cancer by colonoscopy for screening of rectal bleeding. He had a history of chronic hepatitis C. He had no family history. He had no tenderness of the abdomen, and no mass was palpable. Laboratory results were unremarkable. Barium enema showed a filling defect at the rectal ampulla (Figure 1). Colonoscopy revealed a type 2 tumor localized in the lower rectum (Figure 2). Following biopsy, the lesion was confirmed to be papillary adenocarcinoma. Contrast-enhanced CT (CECT) did not reveal swollen lymph nodes or distant metastases but did reveal multiple hypervascular spots in the pancreatic body and tail and abnormally expanded IMV at an arterial phase. During the arterial phase, there was early filling of the portal vein, splenic vein, proximal portion of the superior mesenteric vein, and the abnormally expanded IMV. The IMV, which was approximately 10 mm in diameter, lead tortuously down to the pelvic space and connected to the right internal iliac vein (Figure 3). Angiography of the splenic artery confirmed racemose vascular networks in the body and tail of the pancreas and early venous return to the portal venous system and the abnormally expanded IMV (Figure 4).

Based on these findings, lower rectal cancer with abnormally expanded IMV resulting from P-AVMs was diagnosed. Open distal pancreatectomy was first performed to cure the P-AVMs and thus normalize the abnormally expanded IMV. Surgery revealed a web-like vascular network in the pancreatic body and tail. The IMV was expanded approximately 10 mm in diameter. The IMV poured into the superior mesenteric vein. We performed distal pancreatectomy, preserving the IMV. The splenic artery was cut at the root, and splenectomy was performed immediately. The amount of blood loss was 1,862 ml, and the operation time was 315 min.

**Figure 5:**
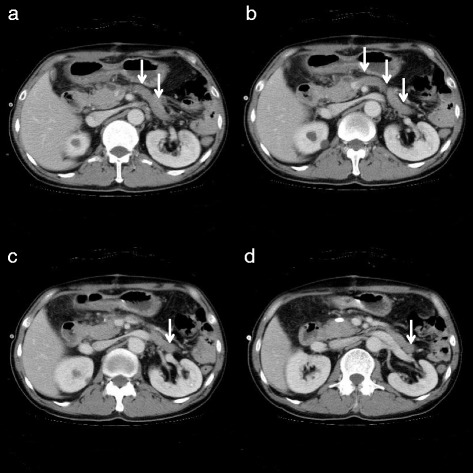
**CECT on POD17 showed occlusion of the abnormally expanded IMV by the thrombi (arrow). (a, b)** The level in which IMV poring into superior mesenteric vein. **(c, d)** Occluded IMV in lower level.

**Figure 6:**
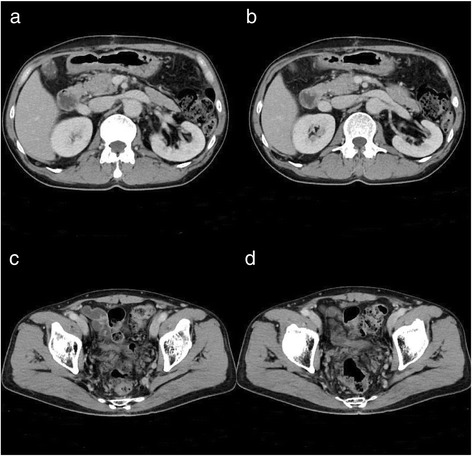
**CECT on POD89 showed no abnormal vessels. (a, b)** The level in which IMV poring into superior mesenteric vein. **(c, d)** Pelvic space.

After the first operation, the patient developed pancreatic fistula (International Study Group of Pancreatic Fistula Grade A), which improved after conservative treatment. CECT showed occlusion of the abnormally expanded IMV by thrombi on postoperative day (POD) 17 (Figure 5). He underwent two courses of preoperative chemotherapy with the CapeOX regimen for 21 days (oxaliplatin: 130 mg/m^2^ drip infusion day 1, capecitabine: 2,000 mg/m^2^ oral administration days 1 to 15) from POD 43. CECT showed no abnormal vessels in his abdomen on POD 89 (Figure 6). We performed radical surgery for rectal cancer on POD 105.

We did not find any abnormal vessels during the second operation. We performed laparoscopic abdominoperineal resection with proximal D3 lymph node dissection for rectal cancer. The few adhesions in his abdomen were easily dissected. The amount of blood loss was 75 ml, and the operation time was 353 min. Histological examination showed papillary adenocarcinoma with a villotubular adenoma component and rectal ulcer scarring. Histological assessment revealed a grade 2 therapeutic effect for nonsurgical cancer therapy. His postoperative course was good except for surgical site infection of the perineal lesion, and he was discharged on POD 42 after the second operation.

## Discussion

P-AVM is a rare disease that was first reported in 1968 by Halpern et al. [2]. The vascular anomaly is present in the pancreas, in which blood flows from the arterial system directly into the portal venous system, without passing through the capillaries in the pancreas. Some patients have a clinical presentation such as epigastralgia and gastrointestinal bleeding; others are asymptomatic [1]. The diagnosis is usually confirmed by angiography and CECT. The angiographic characteristics of P-AVM may include the following: (1) dilated and tortuous feeding arteries from the splenic artery, gastroduodenal artery, or small pancreatic arteries; (2) an intratumoral vascular network followed by a transient dense pancreatic staining due to contrast enhancement or hypervascularity in imaging studies; (3) early filling of draining veins such as the portal vein at the arterial phase; and (4) early disappearance of the pancreatic stain [1]. The typical CT features are the presence of a conglomeration of strong nodular staining and early enhancement of the efferent drainage veins during the arterial phase [3].

Surgical resection, transarterial embolization (TAE), and irradiation are reported to treat P-AVMs [4-8]. In 2013, Chou et al. reviewed the literature and stated that the most common treatment for P-AVM was surgery (43.8%), followed by TAE (11.2%), combination of surgery and TAE (10.1%), and radiotherapy (2.2%) [1]. In our case, abnormally expanded IMV resulting from P-AVMs was revealed by staging CECT for rectal cancer. To the best of our knowledge, this is the first report of P-AVMs combined with rectal cancer. We could not find any case report published in English in PubMed using the following keywords, pancreatic arteriovenous malformation and rectal cancer, pancreatic AVM, and rectal cancer.

The IMV was abnormally expanded throughout its length. It led into the pelvic space tortuously and was connected to the right internal iliac vein. One-stage surgery for rectal cancer was considered to be dangerous because of the high risk of massive intraoperative bleeding. Furthermore, the contracted pelvis makes it difficult to stop the bleeding. Therefore, we decided to cure P-AVMs first and thus normalize the abnormally expanded IMV. Distal pancreatectomy was considered to be most feasible in our case because, although there were multiple AVMs, they were located in the pancreatic body and tail. It was clear from CECT and angiography that the feeding arteries of the P-AVMs were branches from the splenic artery. Although TAE was considered for treatment of the P-AVMs in our case, distal pancreatectomy is a better treatment to reduce the blood flow to the abnormally expanded IMV. The failure rate of TAE is reported to be 37% and additional treatment is needed, including surgery [2,9-11]. Song et al. state in their review that TAE is effective if there is a single apparent feeding artery [6]. If the P-AVMs are multiple, TAE is thought to be difficult [12].

In the initial operation, we performed open surgery because we predicted that manipulation of the area around the pancreas would result in bleeding. Indeed, the tissues around the pancreas bled easily; the amount of bleeding was 1,862 ml. We preserved the abnormally expanded IMV because of the following reasons. First, the abnormally expanded IMV is thought to become thin if the feeding P-AVMs are resected and blood flow is reduced. Second, ligation of the abnormally expanded IMV can lead to blood stasis in the IMV, so the IMV does not become thin. We did not perform ligation or embolization of the IMV in the first operation. As a result, surgical treatment was successful and we could perform safely radical laparoscopic surgery for rectal cancer.

In our case, the abnormally expanded IMV remained after the first operation. Although the IMV was thought to become thin because of the reduction in blood flow, there was no supporting evidence. Intraoperative Doppler or blood gas analysis of the abnormally expanded IMV could have confirmed the efficacy of the treatment.

We added preoperative chemotherapy for the following reason. After the initial operation, although the IMV was occluded by the thrombi on POD 17, we did not know at that time how long it would take for the IMV to become thin. While waiting for the IMV to become thin, we considered that a rectal cancer treatment such as chemotherapy would be helpful. Furthermore, the operation would be easier if the tumor were smaller because it was located in the lower rectum.

The second operation involved laparoscopic surgery because this procedure provides a better view, especially in the intrapelvic space. Lateral lymph node dissection was not performed because the lymph nodes were not enlarged in preoperative CT.

## Conclusions

We have described a patient with rectal cancer and abnormally expanded IMV resulting from P-AVMs, which is believed to be the first case worldwide. Our case revealed the efficacy of surgery for P-AVMs.

## Consent

Written informed consent was obtained from the patient for publication of this case report and any accompanying images. A copy of the written consent is available for review by the Editor-in-Chief of this journal.

## Abbreviations

CT: Computed tomography
CECT: Contrast-enhanced CT
IMV: Inferior mesenteric vein
P-AVM: Pancreatic arteriovenous malformation
POD: Postoperative day
TAE: Transarterial embolization
